# Supplementary material for: Are carbon emissions trading and green financial instruments synergistic? -Comprehensive quantitative research based on content analysis
Source: PLoS One. 2024 Mar 7;19(3):e0298601. doi: 10.1371/journal.pone.0298601 (PMC10919601; doi:10.1371/journal.pone.0298601)
Supplement: S1 Data — (ZIP) [file pone.0298601.s001.zip › Data/地级市Z_F工作报告.zip!/║╙─╧/┐¬╖Γ╩╨/└┤╫╘╣1⁄2╓┌║┼í╛┬φ┐╦╩2╛▌═°í┐_2008.docx]

各位代表：

　　现在，我代表市人民政府，向大会作工作报告，请予审议，并请各位政协委员提出意见。

　　一、2007年政府工作回顾

　　2007年，市人民政府在省委、省政府和市委的正确领导下，高举中国特色社会主义伟大旗帜，以邓小平理论和“三个代表”重要思想为指导，全面落实科学发展观，积极构建和谐社会，团结带领全市人民抢抓机遇，开拓创新，圆满完成了市十二届人大四次会议确定的各项目标任务。

　　——经济总量再上新台阶。2007年全市生产总值达到571.1亿元，比上年增长13.1%，其中一、二、三产业增加值分别增长6.5%、15.5%、15.9%。全部工业增加值达到219.7亿元，增长16.7%，其中规模以上工业增加值完成131.1亿元，增长22.2%；实现利润56.3亿元，增长97.7%，为历年来最高。财政一般预算收入完成20.3亿元，增长24.9%，其中市本级完成8.55亿元，增长19%。

　　——投资、消费和出口快速增长。全社会固定资产投资完成220.3亿元，增长32.5%，其中城镇固定资产投资完成164.1亿元，增长32.4%；社会消费品零售总额209.1亿元，增长17.8%；外贸出口9204万美元，增长21.7%。

　　——财政收入质量明显提高。全市完成税收13.7亿元，增长42.9%，比上年提高30.9个百分点，增幅居全省第一；税收占财政一般预算收入的比重达到67.5%，比上年提高8.5个百分点。

　　——城乡居民收入大幅增加。全市城镇居民人均可支配收入9693元，增长17%；农民人均纯收入3763元，增长18.6%。年末城乡居民储蓄存款余额292.9亿元。

　　——社会事业全面进步。基本普及九年义务教育、基本扫除青壮年文盲工作，代表河南省顺利通过国家验收；科技进步贡献率进一步提升；公共卫生体系不断健全；文化事业快速发展；社会保障体系由城市向农村延伸；人口自然增长率为5‰。

　　这些成绩的取得，标志着我市综合实力和发展后劲明显增强，已经具备了实现跨越式发展的基础和条件。一年来，我们主要做了以下工作：

　　(一)强力实施工业强市战略，工业经济发展实现新突破。园区建设取得新进展，杏花营高新技术产业区、边村重工业区、汪屯精细化工区基础设施建设全面铺开，入驻项目68个，总投资72.9亿元，已建成投产和新开工项目49个。各县工业园区初具规模。重点企业培育取得明显成效，全市规模以上工业企业达到674家，比上年增加118家；销售收入超亿元的企业发展到111家，比上年增加27家，其中开封供电公司、烟草公司销售收入超过10亿元，千里机械、空分设备、宏达拨叉等10家企业销售收入超过5亿元；16家重点工业企业的支撑和带动作用初步显现。产业优势进一步增强，机械设备制造、纺织、食品、化工和医药五大产业完成工业增加值81.2亿元，占全市规模以上工业增加值的比重达到61.9%，比上年增加近10个百分点。重点项目建设进展顺利，82个重点工业项目完成投资52.6亿元，其中40个重点工业项目竣工投产，投资45亿元的京源公司2×600MW超临界发电机组项目正式启动。企业上市实现零的突破，开封高压阀门有限公司成为我市首家上市企业。

　　(二)大力发展农村经济，新农村建设扎实推进。农业生产再获丰收，全年粮食总产244.8万吨，增长4.7%，再创历史新高；肉、蛋、奶总产分别达到31.2万吨、21.7万吨和17.4万吨，畜牧业生产综合评定居全省第四位。县域经济实力进一步增强，五县生产总值达到441.7亿元，其中尉氏县、杞县生产总值突破100亿元；五县财政一般预算收入均超过亿元，其中尉氏县达到2.65亿元。新农村建设扎实推进，47个示范村和300个整治村发展思路更加清晰，村容村貌明显变化。

　　(三)突出发展文化旅游业，第三产业支撑作用不断增强。文化产业亮点纷呈，成功举办了中国开封第25届菊花花会暨首届中国收藏文化(开封)论坛；申办2010年第10届中国菊花博览会取得圆满成功；推出了《大宋·东京梦华》大型水上实景演出和歌舞剧《清明上河图》青春版；发行了《东京梦华·盛世经典》个性化邮票。旅游业发展迅速，接待国内外游客2254.5万人次，实现旅游总收入72.2亿元，分别增长19.2%和19.3%，为近年来最好水平。住宿、餐饮、会展等文化旅游基础设施进一步改善，开元名都大酒店、中州国际酒店、东京艺术中心等一批高档酒店和文化场馆相继建成开业。商贸、邮政、电信、交通运输和房地产业得到较快发展。2007年全市第三产业增加值达到177.7亿元，增长15.9%，拉动经济增长4.8个百分点。

　　(四)继续保持投资快速增长，投资结构明显改善。在国家宏观调控政策趋紧，我市高速公路建设项目相继完成的情况下，我们适时调整投资重点，集中实施了一批农业、能源和工业结构调整项目，保持固定资产投资对经济增长的拉动作用。工业投资首次突破百亿元，全年完成投资108.9亿元，增长93.9%，比上年提高18个百分点。投资结构明显改善，工业投资占城镇固定资产投资的比重达到66.4%，比上年提高21.1个百分点，拉动投资增长42.6个百分点。工业投资的高速增长，进一步增强了我市经济发展的后劲。

　　(五)积极推进改革开放，招商引资成效显著。企业改革不断深入，空分集团由永煤集团全资控股，开化集团炼锌厂、中科印务公司等6家企业完成了产权制度改革，针织内衣厂、汴京机械厂等9家企业正在依法实施破产。非公有制经济持续增长，全年完成增加值323亿元，占全市生产总值的比重达到56.6%。金融体制改革取得重大突破，市农信社用足央行货币政策，为我市争取国家补助资金9.5亿元、企业解困资金16亿元；市商业银行与省投资集团等单位合作重组协议正式签订。城市管理体制不断创新，“两级政府、三级管理、四级网络”的运行模式基本建立。农村公路管理养护体制改革、农村综合改革顺利推进。教育、科技、文化、卫生等领域改革取得了新的进展。招商引资成效显著，引进500万元以上项目510个，总投资251亿元；内资到位147亿元，其中省外资金到位104亿元，居全省第六位；合同利用外资2.5亿美元，实际利用外资5207万美元，增长65%，增幅居全省第三位。尤其是先后引进了海马汽车配件、美国ETQ机电工业及配套、马来西亚大型综合购物广场和杞县聚甲醛等一批投资几十亿元的重大项目，改变了我市长期以来缺少重大项目的状况，这些项目建成后，必将对我市经济发展产生重大影响。

　　(六)加快汴西新区建设步伐，郑汴一体化取得实质性进展。成立了汴西新区管委会，进一步理顺了管理体制。依据《郑汴产业带总体规划》，突出抓好汴西新区中心区8.5平方公里规划编制工作。编制完成了汴西新区水系总体规划、基础设施综合规划以及郑汴产业带开封段村庄整合并迁规划。市发投公司与省投资集团、郑州百瑞信托公司共同出资，成立了开封市基础设施建设开发有限公司，为新区建设搭建了投融资平台。汴西新区基础设施建设全面启动，宋城路西延、1—6号路部分路段建成通车；郑汴产业带开封段入驻和选址项目24个，总投资304亿元，其中郑开森林半岛商住综合楼项目已经奠基，麦德龙商贸物流中心等项目即将开工。阎砦、毛砦村庄整合并迁工程正式启动。

　　(七)全面发展各项社会事业，和谐社会建设取得新成效。组织实施科技计划164项，获国家专利授权180项。继续巩固和扩大“普九”成果，全面推进素质教育，各类学校办学水平进一步提高。科技、文化、卫生“三下乡”活动和社区文化活动深入开展。群众性体育活动和竞技体育健康发展，成功举办了首届郑汴国际马拉松赛和市第七届运动会。认真组织开展了产品质量和食品药品安全专项治理。切实加强环境保护和节能减排工作，关闭取缔重污染和落后产能企业35家，化学需氧量、二氧化硫排放量分别比上年削减7.2%和12.9%；大气质量明显改善，城市空气质量优良天数达到323天。军政军民团结不断加强，再次荣获全国双拥模范城荣誉称号。创“三城”工作取得阶段性成果，我市被评为全省创建工作先进城市。高度重视人民群众来信来访，积极开展社会治安综合治理，夯实公安基层基础，深入开展严打斗争和打击“两抢一盗”犯罪活动，社会治安明显好转。全面加强安全生产管理，安全生产形势总体平稳。自觉接受人大法律监督和工作监督，主动加强与人民政协的联系，广泛听取各民主党派、工商联、无党派人士和人民团体的意见。认真办理人大议案、代表建议和政协提案，全年共承办省、市人大议案、代表建议和政协提案1005件，办复率100%，满意率94.9%。认真落实党风廉政建设责任制，反腐败斗争不断深入。民族、宗教、统计、审计、外事、侨务、供销、人防、气象、广播电视、新闻出版、防震减灾等各项工作都取得了新的成绩。

　　(八)高度关注民生，向人民群众承诺的十五件实事全部落实。坚持公共财政向民生倾斜，切实解决群众在生产生活中的实际困难和问题。认真落实各项惠农政策，发放粮食直补和综合直补资金2.2亿元，直接受益农户93.9万户；切实改善农村生产生活条件，新改建农村公路1154公里，基本实现所有行政村通油路，新建农村户用沼气3.2万座，解决了16.9万人的农村饮水安全问题；认真执行“两免一补”政策，落实资金1.2亿元，受益学生110万人次，继续对城区困难家庭学生免杂费和免费提供教科书；提高了市属中小学公用经费标准，市属中小学公用经费分别由20元/生年、12元/生年提高到100元/生年和60元/生年；加强公共卫生体系和卫生基础设施建设，新增通许县、杞县、金明区三个新农合试点县(区)，农民平均参合率达到80.5%，开工建设了市第一中医院病房楼；进一步繁荣农村文化事业，为农民放映电影4000场，建成了开封县图书馆新馆；实施农村劳动力技能就业计划、农村劳动力培训“阳光工程”，免费培训农村劳动力5.2万人，完成劳务输出115万人次，实现劳务收入63.9亿元；完善和落实就业再就业优惠政策，多渠道开发就业岗位，新增就业6.8万人，实现下岗失业人员再就业3.3万人，其中“4050”人员1.1万人，零就业家庭问题得到及时解决；提高城镇最低工资标准，城镇最低工资由320元/月提高到550元/月；加大对城乡困难群体的救助力度，农村五保户集中供养率提高到31.9%，农村低保由每人每月20元提高到30元，完成脱贫人口3.1万人；新开工建设经济适用房36万平方米，竣工35.3万平方米，为3000户特困家庭发放了廉租住房补贴；改造城市背街27条，为110多条背街安装了路灯，基本消除了城市背街无路灯现象；新建垃圾中转站4座、水冲式公厕10座，更新改造了部分道路环卫设施；投资700万元，调整新增5条公交线路，新增加公交车32台；惠济河、贾鲁河出境断面水质达到省定目标要求，各县污水处理厂和市垃圾处理场基本建成。此外，投资500万元，疏浚了东护城河和东干渠；投资650万元，对鼓楼街、寺后街进行了改造；为2954辆出租车发放燃油补贴676.8万元；为12.5万户养殖户(场)发放能繁母猪和奶牛补贴2246.2万元；为3598名农村计划生育家庭60岁以上老人发放奖励扶助金216万元；为社区工作人员提高了生活待遇，人均生活补贴由200元/月提高到600元/月。通过这些好事、实事的办理，缓解了群众住房难、出行难和就业难、就医难、就学难等问题，城乡居民的生活环境和生活质量也得到了较大改善。

　　各位代表，过去的一年，是我市近年来发展步伐最快的一年，发展质量和经济效益最好的一年，人民群众得到实惠最多的一年。这些成绩的取得，是市委正确领导的结果，是市人大、市政协大力支持和帮助的结果，是全市人民齐心协力、共同奋斗的结果。在此，我代表市人民政府，向全市人民和各民主党派、工商联、无党派人士以及社会各界，向关心、支持开封发展的同志们、朋友们表示衷心的感谢！

　　在肯定成绩的同时，我们也清醒地认识到，我市经济社会发展中还存在不少问题，政府工作中还存在不少薄弱环节。一是经济运行中一些突出问题和深层次矛盾依然存在。工业经济虽然经过一定时期的恢复性增长，具备了由老工业基地向新型工业化城市跨越的基础条件，但规模偏小、发展方式粗放的问题仍然比较突出；第三产业虽然发展势头良好，增速高于全省平均水平，但总量仅占全省的4%；经济结构不合理，二、三产业增加值占生产总值的比重仍低于全省平均水平11.2个百分点；县域经济总体实力不强，农村经济发展不平衡，农民持续增收难度较大。二是涉及群众切身利益的诸多问题有待进一步解决。当前物价上涨是广大群众最为关注的问题，特别是食品和商品房价格涨幅过快，对群众特别是低收入群众生活影响较大，需要我们更加关心。同时，教育、就业、社会保障、医疗卫生、社会治安等方面都有不少问题需要下大力气予以解决。三是政府自身建设和管理需要加强。政府职能转变和机关作风建设与形势发展的要求还有一定差距，部分政府工作人员依法行政观念不强，工作效率不高，形式主义、官僚主义问题依然存在。对这些矛盾和问题，市政府将高度重视，采取有效措施，认真加以解决。

　　二、2008年政府工作的总体要求和目标任务

　　今年是全面贯彻党的十七大精神的第一年，也是我市加快实现“三个跨越”的重要一年。当前，我们既面临着经济和社会发展的重要战略机遇期，同时，由于国内外经济形势不确定因素增多，又面临着更大的压力和挑战。我们必须坚定信心，善抓机遇，坚持又好又快发展的决心不动摇，保护好、引导好、发挥好各方面的积极性，在科学发展、和谐发展、跨越发展的道路上迈出新的步伐。

　　今年政府工作的总体要求是：认真贯彻党的十七大和市委九届五次全会、市委经济工作会议精神，全面落实科学发展观，进一步解放思想，转变作风，坚定不移地实施工业强市、开放引进、旅游带动、科教兴汴、城市化和可持续发展战略，以实现经济跨越式发展为目标，以改革开放和科技进步为动力，着力优化经济结构，提高经济发展质量，更加注重节能减排和生态环境保护，更加重视改善民生和促进社会和谐，为建设富裕、民主、文明、和谐的新开封奠定坚实基础。

　　今年我市经济和社会发展的主要预期目标是：经济增长13%以上，其中一产增长5%，二产增长16%以上，三产增长16%左右；财政一般预算收入增长18%，其中市本级增长18%，达到10亿元；全社会固定资产投资增长25%，其中城镇固定资产投资增长26%；社会消费品零售总额增长17%；进出口总额增长16%，实际利用外资增长30%；控制物价上涨，力争居民消费价格涨幅略低于上年实际水平；城镇居民人均可支配收入增长8%，农民人均纯收入增长6%；新增城镇就业6万人以上，城镇登记失业率控制在4.5%以内；人口自然增长率控制在6‰以下；单位生产总值能耗降低4.5%；化学需氧量、二氧化硫排放量分别下降5.5%和2%。完成上述目标，要重点抓好十个方面的工作：

　　(一)突出发展工业经济，推进产业结构优化升级。坚持以工业化为核心，加大工业投资，膨胀工业规模，提高工业竞争力，争取工业发展速度超过全省平均水平。着力培育骨干企业。加大对空分集团、晋开公司、东大化工、高压阀门等24家重点企业的支持力度，完善动态管理和激励机制，在技术改造、技术创新、要素供给、外部环境等方面给予重点倾斜，支持企业按照市场需求和发展规划扩大投资和生产规模，尽快培育一批大型企业或企业集团，力争今年再有2—3户企业实现销售收入超过10亿元。支持特耐集团、平原非标、新大新科技等公司加快上市步伐。大力培育优势产业。突出抓好机械设备制造、纺织、食品、化工、医药等优势产业发展，着力打造中原城市群特色工业基地。依托龙头企业，整合空分设备、阀门、预应力等行业的生产能力和资源，促进机械行业做大做强；加快尉氏纺织工业园建设，拉长纺织工业链条，解决有纺无织、产业链条短、产品附加值低的问题；依托中原皓月肉牛综合加工、汇源集团果汁加工、万润肉类加工、禾丰肉鸡加工、华中畜禽肉鸭屠宰加工、杞县大蒜加工等农畜产品加工业，大力发展食品工业；抓好晋开公司百万吨总氨、东大化工有机硅、杞县聚甲醛等重大项目建设，提升化工行业规模和竞争力；积极推进企业战略重组，帮助企业谋划和新上项目，着力解决医药行业规模偏小问题。加快发展园区经济和集群经济。坚持节约集约用地，搞好产业规划，加快基础设施建设，强力推进杏花营高新技术产业区、边村重工业区、汪屯精细化工区、黄龙工业区和尉氏—通许—杞县—兰考纺织产业聚集带建设，加快形成一批辐射带动能力强、特色突出的产业集群和产业基地。重点抓好海马汽车配件、永煤集团大型空分设备、通许电动自行车等产业基地建设。加大工业投资力度。优化投资结构，推进工业结构调整，力争完成工业投资150亿元。突出抓好项目建设，着力推进第一批81个重点工业项目建设，确保年内完成投资53亿元。继续实行市领导联系重点企业和重点项目制度，切实抓好京源公司2×600MW超临界发电机组、永煤集团大型空分设备制造、新大新晶硅电池片切磨专用刃料等重大项目建设，使其尽快形成生产能力。加强企业自主创新能力建设。大力发展高新技术产业，提高企业自主创新能力和高新技术的引进、吸收、消化再创新能力。加大企业研发投入，促进企业工程技术中心建设与升级，吸引大型企业科研中心进驻我市。加强与河南大学等大专院校和科研院所的联系，积极促进产学研结合，加快科技成果转化。支持企业技术改造、设备更新和工艺改进，提高产品科技含量，提升企业核心竞争力。大力实施名牌战略，力争实现国家级名牌产品零的突破，新创省名牌产品6个以上。

　　(二)加快发展县域经济，强力推进农村基础设施建设和村容村貌整治。把解决好“三农”问题作为重中之重，加快发展县域经济，不断深化农村改革，积极促进农业稳定发展、农民持续增收，切实解决农村民生问题。发展壮大县域经济。坚持把发展特色支柱产业作为发展县域经济的关键，从各县区的区位、资源和现有产业基础出发，选准有潜力的产业作为支柱产业重点培育，着力推进县域经济特色化。坚持以县域工业园区为载体，积极推进企业向工业园区集中、生产要素向优势企业集中、人口向城镇集中，推动县域经济发展转型。非公有制经济是县域经济最具活力的增长点，要在税收、投融资、资源使用、人才政策等方面，给予积极支持。大力发展劳务经济，继续实施农村劳动力培训“阳光工程”和技能就业计划、扶贫培训“雨露计划”，完成农村劳动力培训15万人。着力发展县域服务业，努力形成与县域主导产业相协调、区域特色鲜明的生产性服务业体系。切实加强金融和融资服务，积极引导农村信用社等金融机构加大对县域经济的支持力度，促进县域经济全面晋位升级，尽快扭转我市县域经济在全省位次靠后的被动局面。稳定提高农业综合生产能力。落实各项惠农政策，继续抓好粮食生产，扩大地方粮食储备规模，增强粮食调控能力。深入推进种植业结构调整，加快优质农产品产业带建设。加强水利基础设施建设，抓好赵口、黑岗口、柳园口、三义寨四大灌区骨干渠道维护和配套建设，开展贾鲁河、涡河、运粮河等重点排涝河道的治理。进一步完善引黄灌溉用水管理机制，切实保障农业灌溉用水。严格耕地特别是基本农田保护，加大中低产田改造力度。实施农田机井通电工程，为农业生产提供可靠电力保障。支持发展农业产业化。立足我市农产品资源优势，采取综合措施，支持农畜产品加工龙头企业发展，促进农畜产品转化增值，增加农民收入。加快农业服务体系建设，鼓励发展各类农村合作经济组织，壮大农民经纪人队伍，提高农业组织化程度。大力发展现代畜牧业，继续实施养殖富群众、加工富财政“双富”工程，以畜牧业重点县和生猪调出大县建设为重点，推进畜牧业区域化布局、规模化养殖和标准化生产。做好重大动植物疫病防控工作，健全农畜产品市场准入制度，提高农畜产品质量安全水平。加快林业生态市建设。突出抓好以农田防护林、防沙治沙、生态廊道网络、村镇绿化、环城防护林及汴西新区6300亩森林公园等重点林业生态工程建设，完成造林绿化23.5万亩，力争林业生态市建设目标3年内完成。加强农村基础设施建设和村容村貌整治。整合资金，分类推进，切实加强环境卫生综合整治，突出抓好农村基础设施建设，完成年度农村道路、饮水、沼气、卫生、文化、电力、连锁超市、通讯网络等建设任务。从治理农村垃圾、污水等问题入手，对800个行政村进行村容村貌集中整治。

　　(三)大力发展第三产业，推进文化旅游大市向文化旅游强市跨越。以建设国际文化旅游城市和中部地区旅游休闲城市为目标，进一步加大投入，创新机制，理顺体制，整合资源，努力构建特色鲜明、结构合理、功能完善、富有竞争力的现代服务业体系，增强第三产业对全市经济的支撑作用。突出发展旅游业。深化旅游景区(点)管理体制改革，整合旅游资源，组建旅游企业集团，做好清明上河园的股权改革和上市工作。加强旅游基础设施建设，按照宋都古城风貌保护与重现工程详规，启动“城摞城”景观展示和延庆观景区开发、繁塔禹王台景区综合开发等项目建设。加大旅游宣传力度，提高旅游接待水平和服务质量，深度开发旅游产品，拉长旅游产业链条，确保全市旅游接待人数增长10%，旅游综合收入增长13%。大力发展文化产业。加大经营性文化单位转企改制力度，加快文化管理体制改革。继续办好中国收藏文化(开封)论坛。做好《大宋·东京梦华》大型水上实景演出和歌舞剧《清明上河图》青春版的市场化运作，打造夜游开封品牌。大力发展文艺演出、新闻出版、影视传媒、广告会展等文化产业，形成实力较强的产业群体。积极发展文化创意产业，培育文化创意市场，催生新的文化业态，不断创造具有自主知识产权的文化知名品牌。改造提升传统商贸餐饮业。扶持有实力、有市场、有发展前景的传统商贸餐饮企业做强做大，引导有条件的老字号、老品牌转换经营机制，提高服务水平，努力使我市的“百年老店”重现辉煌。抓好沃尔玛超市、马来西亚大型综合购物广场等项目建设，提升开封商业档次与品位。加快发展现代服务业。加强市场体系建设，新扩建城北综合农贸市场、金明瓜果市场(二期)等7个市场。规划建设汴东物流中心、开封粮食物流中心，努力打造一批各具特色的现代物流园区和物流品牌。结合汴西新区建设和老城区改造，统筹规划和发展房地产业，规范房地产市场管理，促进房地产业健康平稳发展。加快发展知识技术密集、产业关联度强的会展、信息和中介服务等新兴服务业，加强金融生态环境建设，完成市商业银行重组工作，积极吸引股份制银行和各类金融机构入驻开封。

　　(四)加快老城区改造步伐，推进城市建设与发展。正确处理新区建设与老城区改造的关系，把更多精力用到城市存量资源的整合挖潜上，用到城市内涵式发展上，不断提高城市规划、建设和管理水平。严肃规划审批。以新的《城乡规划法》实施为契机，严格规划审批管理，落实规划公示制度，规范规划执法行为。全面完成《开封市城市总体规划》修编和老城区改造修复性详细规划，编制完成城中村改造整体规划，启动社区建设专项规划。加快老城区和城中村改造步伐。完善拆迁管理办法，认真做好被拆迁群众的安置工作。完成南城墙迎宾岗至大南门段墙体修缮保护和墙外50米环境整治，对西城墙北段外侧局部地带进行整治；完成水系二期河道及两侧景观绿化工程，同步跟进水系两侧景观建设；结合水系工程建设，启动老城区1号、2号地块改造工程。按照城中村改造规划，启动金明区演武庄村、堌门村，顺河回族区郭屯村，龙亭区孙李唐村、小李庄村以及北关街改造工程。加快城市基础设施建设。完成夷山大街南延至魏都路、集英街北延至解放路、汴京路东延至东五路、工农路北延至新曹路建设工程；对向阳路、晋安路、铁路北沿街进行改造；对中山路(新街口-大南门)、省府前街(四面钟-西司桥)、大庆路等城市主次干道进行景观整治。完善城市防汛设施，建成西环路雨水泵站，改造禹王台立交泵站。配合省有关部门，积极做好郑州至开封轻轨和郑州至民权高速公路建设项目的前期工作。切实加强县城管理，完善县城基础设施，提高县城建设水平。抓好沿国道、省道小城镇的规划与建设，着力抓好朱仙镇的规划、建设和发展，加强政策、资金支持，再现名镇风采。深入开展创“三城”活动。加大城市美化、绿化、亮化、净化力度，突出抓好市容环境、市政设施、园林绿化、公共交通等方面的精细化管理，积极探索城市网格化管理新模式，健全城市管理长效机制，坚决治理城市“八乱”，努力打造中原宜居城市。

　　(五)加快汴西新区建设，推进郑汴一体化发展。积极争取省政府批准郑州、开封为河南省区域协调发展和配套改革试验区，深入推进电信同城、金融同城和旅游、文化、广电等资源的整合共享，集中精力加快中心区域和起步区建设。加快新区基础设施建设。全面启动汴西新区起步区道路建设，加快建设1号路、4号路、5号路(宋城路-郑开大道)和宋城路西延(6号路-11号路)工程，开工建设3号路(汉兴路-郑开大道)、9号路(郑杞连线-郑开大道)、周天路(1号路-6号路)、汉兴路西延(1号路-4号路)、晋安路西延(1号路-6号路)、魏都路西延(6号路-9号路)等工程，同步跟进管网配套设施。加快热源、气源、污水处理、排水泵站等基础工程建设。实施汴西新区水系总体规划，积极做好黑岗口调蓄水库建设的前期工作。稳步推进村庄整合并迁。按照尊重民愿、便民利民、科学规划、高效集约、稳步推进的原则，加快阎砦、毛砦村庄整合并迁。做好汴河堤、横堤铺等村的整合并迁规划，适时启动。有序展开产业布局。继续把项目建设作为新区建设的首要任务，按照郑汴产业带规划要求，做好项目规划、储备和申报，加强与省直有关部门的协调与沟通，争取在土地、资金、项目、政策等方面的支持，积极促进项目落地。对已批准项目，坚持高起点规划、高标准建设，力争把郑汴产业带开封段建设成为国内一流的产业聚集区。

　　(六)加大节能减排和环境保护力度，推进生态文明建设。今年是完成“十一五”节能减排约束性目标的关键一年，必须采取有效措施，力求取得更大成效。强化能源资源节约。认真落实节能减排统计、监测、考核实施方案和办法，严格实行节能减排问责制和一票否决制。严把项目准入关，严禁新上高耗能、高污染项目。坚持结构性节能和工程性节能两手抓，严格执行能耗准入标准，突出抓好年耗能5000吨标煤以上31家企业的节能工作。继续开展土地“三项整治”，巩固砖瓦窑厂关闭成果，加强土地复垦工作。以开发区、工业园区为重点，推广多层标准厂房。加强建筑领域节能，积极推广新型墙材，县以上城市全面禁止使用实心黏土砖。加强环境污染治理。继续抓好重点流域、区域和行业的环境综合整治。编制饮用水源地保护规划。全面推进污水处理厂配套管网建设，确保实现稳定运行、达标排放。实施农村小康环保行动计划，控制和治理农村污染。推动污水、垃圾处理产业化，加快发展环保产业。

　　(七)继续深化各项改革，推进体制机制创新。以纪念改革开放30周年为契机，总结经验，深入推进，努力开创我市改革开放新局面。继续深化企业改革。坚持因企施策，分类指导，引导企业明确发展定位，加快改革步伐。用好比照振兴东北老工业基地政策，以引进战略投资者为重点，以妥善安置职工为前提，积极推进开化集团、联合收割机厂、缝纫机总厂等国有企业改革和空压机厂、汴绣厂等集体企业改革。抓好博达公司、前锋制药公司、轴承公司的二次改革。大力发展非公有制经济。坚持以创业带就业的积极就业政策，开展创业培训，打造创业平台，完善创业服务体系，优化全民创业环境。加快市、县(区)中小企业投资担保和小额贷款担保机构建设，实施小老板培育和中小企业成长工程，促进非公有制经济更好更快发展。深化事业单位人事制度改革。在事业单位分类管理、编制总量控制的基础上，以转换用人机制和搞活内部分配制度为重点，以推行全员聘用制度和岗位管理制度为主要内容，逐步建立政事职责分开、法人地位明确、单位用人自主、政府依法监管、促进事业单位可持续发展的人事管理体制。重点抓好教育、卫生系统事业单位人事制度改革。全面推进其它领域改革。深化服务业体制改革和机制创新。扎实推进农村公路管理养护体制改革，实行县道县养、乡道乡养、村道村养。继续深化社会管理体制、公用事业等领域的改革，全面推进财税、价格、投融资、林权、金融等各项改革。

　　(八)继续扩大对外开放，推进开放型经济发展。着力谋划一批符合开封实际的重大项目，做好项目筛选、包装、论证、储备工作，积极推行规划招商、园区招商、产业招商、定向招商、乡情招商、以商招商，做好项目跟踪服务，努力提高合同履约率、资金到位率、项目成功率，增强招商引资实效。坚持走出去、请进来，积极参加中博会、厦交会等各类经贸活动，办好中国开封第26届菊花花会，把招商引资与外事活动结合起来，拓宽招商引资渠道，提高招商引资水平。优化出口产品结构，积极扩大高新技术产品和高附加值产品出口，提升劳动密集型出口产品档次，巩固化工、机电、纺织等优势产品出口，扩大原材料和关键设备、先进技术进口，完成外贸进出口1.34亿美元。加强对外经济技术交流与合作，承包工程新签合同额和完成营业额分别增长10%和12%。积极开拓境外劳务渠道，强化技能培训，确保境外劳务增长12%。进一步优化发展环境，深入推进行风建设，继续开展窗口行业规范管理优质服务竞赛活动，以环境促开放，以环境优化赢得发展先机。

　　(九)加快发展社会事业，推进和谐开封建设。以解决人民群众最关心、最直接、最现实的问题为重点，加快发展各项社会事业，努力使广大群众学有所教、劳有所得、病有所医、老有所养、住有所居，全面推进和谐社会建设。优先发展教育事业。加大对教育的投入力度，整合优化教育资源，改善办学条件，提高教育质量。全面实施城乡免费义务教育，加快普及高中阶段教育，提高职业教育水平，稳步发展高等教育，支持发展民办教育。积极发展文化体育事业。加快文化馆、图书馆、乡镇综合文化站建设。加强重点文物和非物质文化遗产保护和开发。搞好城镇数字电影广场建设，实施农村地面数字电视“家家通”工程。精心组织好北京奥运会火炬接力开封市传递活动。推进全民健身和竞技体育深入开展。大力发展卫生事业。加快建立覆盖城乡居民的公共卫生服务体系、医疗服务体系、医疗保障体系、药品供应保障体系，为群众提供安全、有效、方便、价廉的医疗卫生服务。建立和完善基本卫生制度，做好城镇居民医疗保险和新型农村合作医疗工作。加强对乡、村医务人员的业务培训，提高农村医疗卫生水平。加强公共卫生体系建设，完善疾病预防和医疗救治工作措施，遏制重大传染疾病的暴发流行。切实加强社会管理。加强社区建设，充实社区工作力量，完善社区服务功能，使社区真正成为群众自我教育、自我管理、自我服务的重要平台。及早部署，精心组织，做好第六届村民委员会换届选举工作。严格产品质量和食品药品安全管理，进一步优化消费环境。落实安全生产责任制，强化安全生产监督和管理，遏制重特大事故发生。做好信访稳定工作，有效预防和化解各类社会矛盾。健全社会治安防控体系，防范和依法打击各类违法犯罪，深入开展平安创建活动。认真贯彻落实党的民族宗教政策，促进各民族群众和谐共处、共同发展。坚持计划生育基本国策，稳定低生育水平，优化人口结构，提高人口素质。加强法律援助，依法保障公民特别是妇女、未成年人和残疾人的合法权益。

　　(十)着力办好十五件实事，推进民生工程建设。政府的一切权力都是人民赋予的。我们要牢记全心全意为人民服务的宗旨，把实现好、维护好、发展好最广大人民的根本利益，作为政府工作的出发点和落脚点，更加关注保障和改善民生，特别要关心和解决城乡低收入群众的生活困难，使全体人民共享改革发展成果。今年，我们要继续办好关系人民群众切身利益的实事、好事，努力在改善民生上取得更大成效。1.加大对农民的补贴力度。继续实施对种粮农民直接补贴、良种补贴、农村劳动力转移培训补贴和测土配方施肥补贴；较大幅度增加农业生产资料综合直补、农机具购置补贴；认真落实肉牛、肉羊、奶牛和生猪生产扶持政策；积极开展家电下乡试点工作，对农民购买国家规定品牌和型号的彩电、冰箱、手机，按销售价格给予13%的直接补贴。2.切实改善农村村容村貌。完成1500个行政村的村庄规划；改造县乡公路310公里；解决18万农村人口的饮水安全问题；新建农村户用沼气2.5万座；完成扶贫开发整村推进13个村，脱贫人口4.4万人；完成“盲点村”电网改造任务；绿化村(镇)800个；新改建农家店500家。3.大力发展农村文化事业。深入开展文化下乡活动，丰富农民业余文化生活；加强农村文化设施建设，建成3-5个乡镇综合文化站；加快推进农村电影放映工程，使60%的行政村实现一村一月放映一场公益性电影。4.进一步提高义务教育保障水平。在城市西区改扩建初中、小学各一所；从今年秋季学期开始全面免除城市义务教育阶段学生杂费；为农村义务教育阶段学生全部免费提供教科书；提高农村中小学生生均公用经费标准，农村小学、初中分别达到230元／生年、375元／生年，县镇小学、初中分别达到245元／生年、390元／生年；提高农村家庭经济困难寄宿生生活费补助标准，小学、初中由每名学生每天1元分别提高到2元和3元；实施农村中小学图书教学仪器设备充实工程；积极解决农民工子女入学和城镇大班额问题。5.继续改善群众医疗卫生条件。在全市农村全面实行新型农村合作医疗制度，农民平均参合率达到85%，参合标准由每人50元提高到90元；完成市紧急救援中心建设，确保年内投入使用；启动西区综合性医院建设；加强艾滋病防治，对定点村卫生室和乡镇卫生院艾滋病救治防治给予经费补贴，落实艾滋病救治防治乡村一线医务人员待遇，提高艾滋病救治水平。6.扎实推进城市低收入家庭住房保障工作。开工建设经济适用房50万平方米，竣工30万平方米；启动廉租住房建设，新建、收购廉租住房2万平方米，提供廉租住房房源400套；提高廉租住房租赁补贴标准，扩大补贴范围，对市区符合条件、申请廉租住房租赁补贴的最低收入家庭做到应保尽保，逐步将保障范围由最低收入家庭扩大到低收入家庭，对各县符合住房保障条件的最低收入家庭基本做到应保尽保。7.认真做好就业再就业工作。全年城镇新增就业6万人以上，实现下岗失业人员再就业2.5万人，安置“4050”人员再就业0.9万人以上；及时解决零就业家庭问题。8.加强社会保障和对城乡困难群众的救助。继续提高企业退休人员基本养老金水平，今年人均增加109元；全面推行城镇居民基本医疗保险，着力解决国有困难企业退休人员医疗保障问题；提高城乡低保标准，城市低保对象人均月补差标准由70元提高到95元，农村低保标准由不低于30元提高到不低于40元。9.加大农村敬老院建设力度。新建农村敬老院6所，改扩建敬老院23所，争取农村五保对象集中供养率达到40%；提高五保供养标准，集中供养五保对象的最低标准由1200元/人年提高到1400元/人年，分散供养五保对象的最低标准由1000元/人年提高到1100元/人年。10.努力保障食品药品安全。健全农畜产品市场准入制度，强化食品、药品质量监管，实施食品放心工程，开展食品安全专项整治，及时查处食品、药品质量安全违法违规案件；鼓励药品连锁和集中配送向农村发展，筹措资金建设农村药品监督网和供应网。11.积极推进污染防治。继续加强惠济河流域污染的专项整治，实现水质明显好转；加强饮用水源地安全建设；建成的城市污水处理厂全部实现稳定运行、达标排放；继续加大对环卫设施的投资力度，加强城镇生活垃圾处理场运营管理。12.加强社区建设。新(扩)建社区办公用房40个，力争年内城区三分之一社区办公用房达到300平方米以上；新建社区卫生服务中心5个、社区卫生服务站20个，年底社区卫生服务覆盖率达到80%以上。13.提高城市公交运营能力。投资1000万元，更新公交车辆53台，新开、更新公交线路5条，进一步解决群众出行难问题。14.提升城市亮化水平。投资1100万元，新建城市照明18.4公里，改建城市照明7公里，基本消除城市道路无路灯现象。15.加快背街改造步伐。投资2245万元，对旧城改造项目以外的115条背街全部进行改造。

　　三、加强政府自身建设，提高依法行政能力和政府工作水平

　　全面落实科学发展观，实现开封经济社会又好又快发展，对政府行政能力提出了更高要求。我们要坚持改革创新，转变政府职能，切实加强政风建设，努力建设服务型政府。

　　(一)依法行政。严格执行人大及其常委会的决议、决定，自觉接受人大及其常委会的法律监督和工作监督；加强与人民政协的协商，自觉接受人民政协的民主监督；广泛听取各民主党派、工商联和无党派人士的意见和建议。依法规范行政行为，深入推进行政执法责任制，健全质询、问责、经济责任审计等制度，严格按照法定权限和程序行使权力、履行职责，确保权力在阳光下运行。畅通行政复议渠道，办好行政复议案件。

　　(二)深化改革。按照中央和省统一部署，积极推进行政管理体制改革，着力转变职能、理顺关系、优化结构、提高效能，形成权责一致、分工合理、决策科学、执行顺畅、监督有力的行政管理体制。进一步健全政府各部门之间的协调配合机制，提高行政效率。深化行政审批制度改革，进一步减少审批事项，简化和规范审批程序，强力推进窗口办结制。

　　(三)改善服务。在加强和改善经济调控、市场监管的同时，更加注重履行社会管理和公共服务职能。完善公共服务体系，优化公共资源配置，切实为社会、企业和人民群众提供优质便捷服务。认真落实政府信息公开条例，保障人民群众的知情权、参与权、表达权和监督权。加强电子政务建设。完善应急预案体系和管理体制，有效应对自然灾害、事故灾难、公共卫生和社会安全突发事件，提高危机管理和抗风险能力。

　　(四)转变作风。牢固树立正确的政绩观，坚持求真务实、真抓实干，增强忧患意识、责任意识、节俭意识、公仆意识，倡导想干事、会干事、干成事的工作作风。健全政府绩效考核体系，把各方面工作引导到科学发展、和谐发展轨道上来。大兴调查研究之风，提高科学决策水平。切实加强效能建设，增强政府公信力和执行力。大力精简会议、文件，切实改进会风、文风，严格控制和规范各种检查、评比、达标活动，力戒形式主义和官僚主义。牢记“两个务必”，严于律己、廉洁奉公、兢兢业业、干干净净为国家和人民工作。坚决反对铺张浪费和奢靡之风，建设学习型、节约型机关。加强反腐倡廉建设，坚决惩治和有效预防腐败。进一步提高公务员管理法制化、规范化水平，努力建设一支政治坚定、业务精通、清正廉洁、作风优良的公务员队伍。

　　各位代表，让开封这座千年古都重现辉煌，是时代赋予我们的光荣使命和神圣职责。让我们紧密团结在以胡锦涛同志为总书记的党中央周围，高举中国特色社会主义伟大旗帜，以邓小平理论和“三个代表”重要思想为指导，深入贯彻落实科学发展观，在省委、省政府和市委的正确领导下，团结奋进，真抓实干，为实现开封跨越式发展而努力奋斗！
